# Dihydrocoumarin, an HDAC Inhibitor, Increases DNA Damage Sensitivity by Inhibiting Rad52

**DOI:** 10.3390/ijms18122655

**Published:** 2017-12-07

**Authors:** Chin-Chuan Chen, Ju-Sui Huang, Tong-Hong Wang, Chen-Hsin Kuo, Chia-Jen Wang, Shu-Huei Wang, Yann-Lii Leu

**Affiliations:** 1 Graduate Institute of Natural Products, Chang Gung University, Taoyuan 333, Taiwan; chinchuan@mail.cgu.edu.tw (C.-C.C.); judyhuang810@gmail.com (J.-S.H.); xeriok70767@gmail.com (C.-H.K.); 2 Tissue Bank, Chang Gung Memorial Hospital, Taoyuan 333, Taiwan; cellww@gmail.com; 3 Chinese Herbal Medicine Research Team, Healthy Aging Research Center, Chang Gung University, Taoyuan 333, Taiwan; 4 Graduate Institute of Health Industry Technology, Research Center for Industry of Human Ecology, College of Human Ecology, Chang Gung University of Science and Technology, Taoyuan 333, Taiwan; 5 Liver Research Center, Chang Gung Memorial Hospital, Linko 333, Taiwan; 6 Institute of Stem Cell and Translational Cancer Research, Chang Gung Memorial Hospital, Taoyuan 333, Taiwan; wangcj225@cgmh.org.tw; 7 Department of Anatomy and Cell Biology, College of Medicine, National Taiwan University, Taipei 100, Taiwan; 8 Center for Traditional Chinese Medicine, Chang Gung Memorial Hospital, Taoyuan 333, Taiwan

**Keywords:** yeast, DSB, DHC, DNA damage sensitivity, homologous recombination, Rad52

## Abstract

Effective DNA repair enables cancer cells to survive DNA damage induced by chemotherapeutic or radiotherapeutic treatments. Therefore, inhibiting DNA repair pathways is a promising therapeutic strategy for increasing the efficacy of such treatments. In this study, we found that dihydrocoumarin (DHC), a flavoring agent, causes deficiencies in double-stand break (DSB) repair and prolonged DNA damage checkpoint recovery in yeast. Following DNA damage, Rad52 recombinase was revealed to be inhibited by DHC, which results in deficiencies in DSB repair and prolonged DNA damage checkpoint recovery. The deletion of *RPD3*, a class I histone deacetylase (HDAC), was found to mimic DHC-induced suppression of Rad52 expression, suggesting that the HDAC inhibitor activity of DHC is critical to DSB repair and DNA damage sensitivity. Overall, our findings delineate the regulatory mechanisms of DHC in DSB repair and suggest that it might potentially be used as an inhibitor of the DNA repair pathway in human cells.

## 1. Introduction

Chemotherapy and radiotherapy are major cancer treatments that generate DNA damage in cancer cells [1,2,3]. However, some malignant cancer cells develop an efficient DNA repair machinery through further mutation, allowing the tumor cells to repair DNA damage induced by radio- or chemotherapeutic treatment [4,5,6,7]. Thus, the inhibition of DNA repair pathways could sensitize the therapeutic effects of radiotherapy or DNA-damaging chemotherapeutic drugs [8,9,10,11]. Indeed, several inhibitors of DNA repair pathways have been used in combination with chemotherapy and/or radiotherapy [12,13].

Radio- and chemotherapeutic drugs generate DNA damage that would cause improper chromosome segregation during cell division if they were not properly repaired, and the most severe DNA damage is double strand breaks (DSBs). DSBs trigger the DNA damage checkpoint, leading to cell cycle arrest to allow time for DNA repair [14,15]. In budding yeast, Tel1 and Mec1 (ATM and ATR in human, respectively) initiate the DNA damage checkpoint [16,17]. Upon their recruitment to DNA, Mec1 and its binding partner Ddc2 (ATRIP in human) phosphorylate a group of targets, including histone H2A. Mec1-Ddc2 subsequently phosphorylates Rad9 to activate Rad53 (CHK2 in human) [16,17], and the activation of Rad53 plays an important role in cell cycle arrest and enables comprehensive DNA repair [16,17,18]. 

Homologous recombination repair (HR) and non-homologous end-joining (NHEJ) are two major mechanisms for the repair of fix DSBs [19,20]. HR is an accurate repair pathway, whereas the NHEJ pathways are less accurate and potentially cause DNA rearrangements [20,21]. In both cancer and budding yeast, HR is the major repair pathway for overcoming DSB lesions [22,23]. HR initiates with extensive 5′ to 3′-end resection at the broken ends, which in yeast is regulated by Mre11/Rad50/Xrs2 (MRN in human) and Sae2 (CtIP in human) [24,25]. Subsequently, replication protein A (RPA) binds the resected DNA and facilitates loading of the recombinase Rad51, a process that is mediated by Rad52 [23,26]. The HR repair process is then completed by DNA synthesis and Holliday junction resolution [23,26,27].

Dihydrocoumarin (DHC) is a compound found in *Melilotus officinalis* (sweet clover) that has been found to inhibit the NAD-dependent histone deacetylase (HDAC) Sir2 in yeast [28]. Because HDAC inhibitors inhibit the DNA damage response (DDR) [29,30], DHC might suppress the DNA repair machinery by inhibiting HDAC activity. However, these activities have not been identified due to the sequence-independent nature of radiation or chemotherapy drug-induced damage. 

In this study, we used an inducible HO endonuclease system [31], which generates a DSB at a specific site that can be repaired by single-strand annealing (SSA), one variant of HR. Using the SSA system, the results revealed that DHC sensitizes yeast cells to DNA damage by regulating Rad52 and influences damage-induced apoptosis and autophagy. Our results support the notion of targeting DNA repair with DHC, which could provide a valuable model for identifying the effects of the combined use of DHC and radio- or chemotherapy.

## 2. Results

### 2.1. DHC (Dihydrocoumarin) Inhibits Double-Strand Break Repair

**Figure 1:**
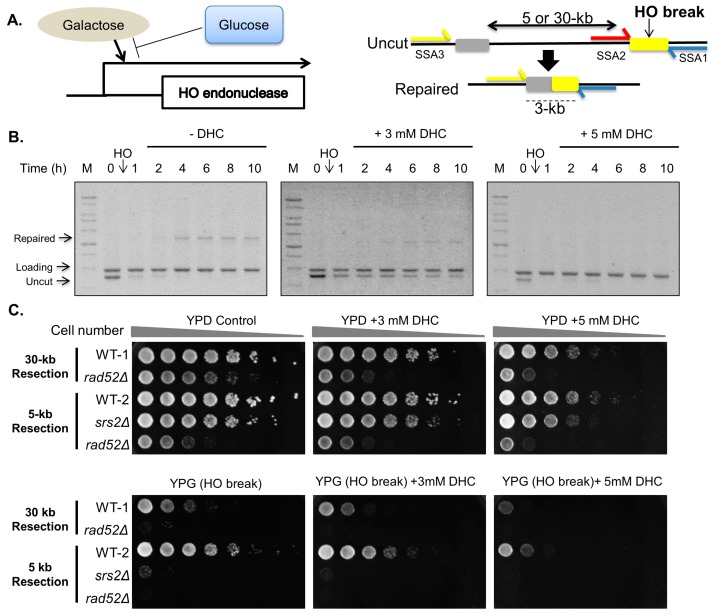
DHC increases DNA damage sensitivity and inhibits DNA double-strand break repair in SSA strains. (**A**) Schematic diagram of the single strand annealing (SSA) system. Galactose was used to induce HO endonuclease and thus generate a specific HO lesion. Repair of the HO lesion at the HO cleavage site (yellow box) requires a 5-kb or 30-kb resection back to the uncleavable HO cleavage site (gray box). Three polymerase chain reaction (PCR) primers (SSA1, SSA2 and SSA3 primers) were used for measuring DNA damage and repair; (**B**) DHC inhibits DNA double-strand break repair in the 5-kb resection strains (YMV045). The DSB was induced by the addition of galactose to the YMV045 strain. After 30 min, the cultures were treated with 3 mM or 5 mM DHC, and the repair of the HO lesion was analyzed by PCR. The marker is labeled M; (**C**) Five-fold serial dilution analysis of WT-1 (YMV002), *rad52∆* (YMV037), WT-2 (YMV045), *srs2∆* (YMV057), and *rad52∆* (YMV046) shows sensitivity to galactose and DHC. The cells were allowed to grow at 30 °C for 3 days and were photographed for recording colony formation.

We first investigated whether DHC inhibits the DNA repair machinery. Because DNA DSBs are generated at unpredictable locations after DNA damage agent treatment, it is hard to address DNA repair in these sites following such treatments. Here, we used an HO endonuclease-mediated system (SSA strain) to generate a specific DSB, which allowed us to investigate the mechanism of DNA repair (Figure 1A) [31]. The SSA system contains an uncut region homologous to the HO site. Following the induction of an HO break, the 5′ ends are resected, exposing the single-strand DNA such that the complementary strands can anneal to each other to generate a 3-kb repair product. Thus, the repaired products can be monitored by PCR. Through PCR analysis, we found that DHC inhibits DSB repair in a dose-dependent manner (Figure 1B). SSA strains were plated onto glucose plates (yeast extract-peptone-dextrose, YPD) or galactose plates (yeast extract-peptone-galactose, YPG) to induce an HO lesion with the aim of testing whether DHC sensitizes SSA strains to a DSB. Rad52 mutants were used as a negative control, and these failed to repair the HO lesion. We observed that wild-type cells of either 5-kb or 30-kb resection strains were slightly sensitive to DHC (YPD + DHC) but became more sensitive to DHC containing an HO lesion (YPG + DHC) (Figure 1C). These results indicate that DHC sensitizes SSA strains to a single DSB and suggest that DHC enhances DNA damage sensitivity by inhibiting DSB repair in yeast.

### 2.2. DHC Sensitizes Yeast Cells to DNA-Damaging Drugs

**Figure 2:**
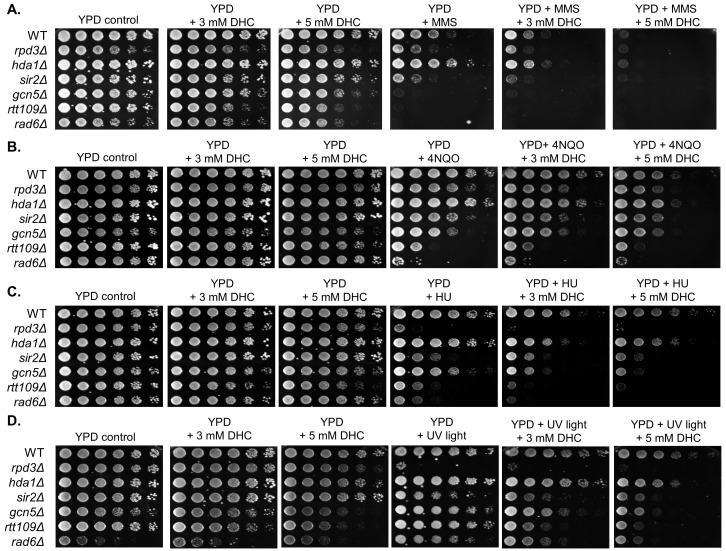
DHC sensitizes wild-type cells to DNA-damaging drugs. Five-fold serial dilution analysis of the indicated isogenic strains, including WT (BY4741), *rpd3∆* (BY4741-rpd3), *hda1∆* (BY4741-hda1), *sir2∆* (BY4741-sir2), *gcn5∆* (BY4741-gcn5), and *rtt109∆* (BY4741-rtt109), shows their sensitivity to: (**A**) 0.02% MMS; (**B**) 0.1 µg/mL 4NQO; (**C**) 100 mM HU; and (**D**) 150 J/m^2^ UV light with or without DHC. The cells were allowed to grow at 30 °C for 3 days and were photographed for recording colony formation.

We then investigated whether DHC increases DNA damage sensitivity following treatment with DNA-damaging drugs in yeast. By plating wild-type and control strains onto YPD plates containing DHC, DNA-damaging drugs or DHC plus DNA-damaging drugs, we found that *rad6*, *rtt109*, *gcn5*, *rpd3* (Class I HDAC) and *sir2* (Class III HDAC) control mutant yeast cells were highly sensitive to DNA-damaging agents, which is similar to previous findings (Figure 2) [32,33]. We also found that the growth of wild-type cells was slightly affected by DHC or DNA-damaging drugs, but wild-type yeast became extremely sensitive to methyl methanesulfonate (MMS, a DNA alkylating agent that generates single- and double-strand breaks) in combination with DHC. Moreover, the degree of sensitivity corresponded with the dose of DHC (Figure 2). In addition, DHC just slightly sensitized wild-type cells to 4-nitroquinoline-1-oxide (4NQO, an ultraviolet-mimetic agent that generates cross-linked DNA), UV light and hydroxyurea (HU, a replication-dependent damaging drug) (Figure 2). 

### 2.3. DHC Postpones DNA Damage Checkpoint Recovery

**Figure 3:**
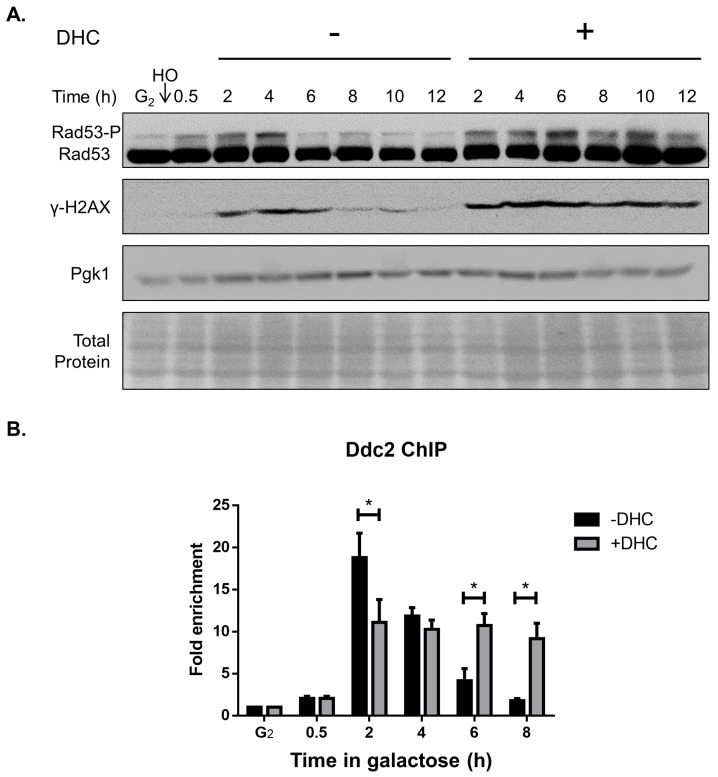
DHC influences the DNA damage checkpoint. (**A**) Wild-type cells (YMV045) were arrested at G2 with 15 µg/mL nocodazole, and HO endonuclease was induced by the addition of galactose to generate a DSB. After 30 min, the cultures were divided equally and treated with or without 5 mM DHC. Anti-Rad53 and anti-Y-H2A antibodies were used for the detection of protein expression by immunoblotting. Amido black staining of the total protein and Pgk1 protein levels, which served as the loading controls, was performed; (**B**) The recruitment of Ddc2 to flank the HO lesion in *DDC2::MYC* cells (RLY001) was analyzed by ChIP. The error bars represent the standard deviations from at least three independent experiments. Asterisks (*) indicate significant differences between DHC-treated and untreated cells (* *p* < 0.05).

Activation of the DNA damage checkpoint is important for DSB repair because it arrests the cell cycle to avoid mitosis with damaged DNA [20,34]. We questioned whether DHC affects the DNA damage checkpoint following a DSB, and our immunoblotting results showed that Rad53 and H2A were continuously phosphorylated following DHC treatment after a DSB, revealing that DHC postponed the G_2_/M DNA damage checkpoint (Figure 3A). We subsequently investigated the mechanism through which DHC influences the G_2_/M DNA damage checkpoint. The recruitment of Ddc2 to DSBs is a crucial step for activating the DNA damage checkpoint [35]. We thus determined whether DHC affects the recruitment of Ddc2 to a DSB through a chromatin immunoprecipitation (ChIP) analysis. In the absence of DHC treatment, Ddc2 was recruited to a DSB shortly after induction of an HO lesion and remained for 6 h (until the DSB was repaired, which is known as checkpoint recovery). However, with DHC treatment, the recruited Ddc2 persisted for more than 8 h (Figure 3B). This result demonstrated that yeast cells remain stuck at G_2_/M when DSB repair is deficient. 

### 2.4. Rad52 Is Inhibited by DHC in Response to a DSB (Double-Stand Break)

**Figure 4:**
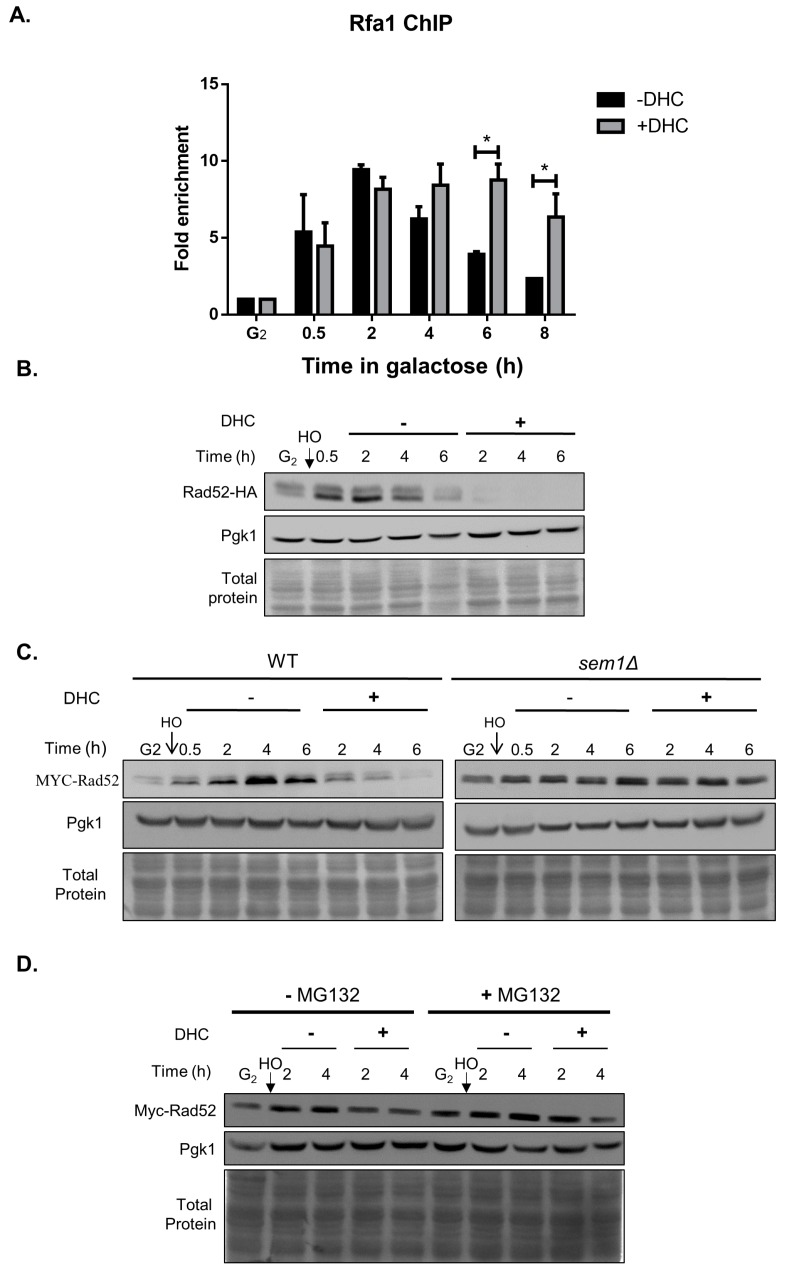
Rad52 protein expression is inhibited by DHC following DNA damage. (**A**) *RFA1-MYC* cells (YAY022) were arrested at G2 with 15 μg/mL nocodazole, and HO endonuclease was induced by the addition of galactose to generate a DSB. After 30 min, the cultures were divided equally and treated with or without 5 mM DHC. The recruitment of Rfa1 to flank the HO lesion was analyzed by ChIP. The error bars represent the standard deviations from at least three independent experiments. Asterisks (*) indicate significant differences between DHC-treated and untreated cells (* *p* < 0.05); (**B**) *RAD52-HA* cells (YAY013) were arrested at G2 with nocodazole, and HO endonuclease was induced by the addition of galactose to generate a DSB. After 30 min, the cultures were divided equally and treated with or without 5 mM DHC. Whole-cell extracts were analyzed by immunoblotting at the indicated time points; (**C**) The WT (*RAD52::MYC*) cells (YAY028) or *sem1* mutant cells (RLY006) were arrested at G2, and HO endonuclease was induced by the addition of galactose at time 0 to generate a specific DSB. The culture was then split into two groups, namely, −DHC (WT) and +DHC (5 mM DHC), and samples were then processed for immunoblotting analysis; (**D**) DHC inhibits Rad52 recombinase protein levels after MG132 treatment following DNA damage. *RAD52::MYC* cells (YAY028) were arrested at G2 with nocodazole, and HO endonuclease was induced by the addition of galactose at time 0 to generate a specific DSB. The culture was then split into four groups: −DHC (5 mM) −MG132 (1 mM), +DHC −MG132, −DHC +MG132 and +DHC +MG132. Samples collected at the indicated time points were then processed for immunoblotting analysis. The Pgk1 protein level and total protein, as assessed through amido black staining, served as loading controls.

In response to DSBs, HR repair requires DNA resection, which allows RPA to bind to single-strand DNA (RPA-ssDNA) [36]. We performed a ChIP analysis to measure the recruitment of Rfa1, a subunit of RPA, to ssDNA in the vicinity of an HO lesion in the presence or absence of DHC treatment to identify whether DHC treatment inhibits DNA resection. We observed that DHC treatment does not inhibit Rfa1 recruitment but rather prolongs the binding of Rfa1 to damaged DNA (Figure 4A). Because homologous recombination is the next main step of DSB repair after DNA resection, the data suggest that DHC inhibits homologous recombination, which accumulates high amounts of ssDNA that binds with RPA. We investigated whether the recombination protein Rad52, which is essential for HR repair in yeast, was affected by DHC in response to a DSB by analyzing the protein expression levels of Rad52 and found that DHC reduced Rad52 protein expression after HO induction (Figure 4B). We then examined how DHC inhibits Rad52 protein expression using an *SEM1* depletion strain that lacks the 26S proteasome regulatory subunit and found that DHC failed to inhibit the protein levels of Rad52 in *sem1* mutants by (Figure 4C). Similar results were obtained with the treatment of yeast with MG132, which inhibits the proteasome degradation of ubiquitin-conjugated protein. The protein levels of Rad52 were rescued by MG132 following DHC treatment (Figure 4D). These data suggest that DHC promotes Rad52 degradation post-translationally.

**Figure 5:**
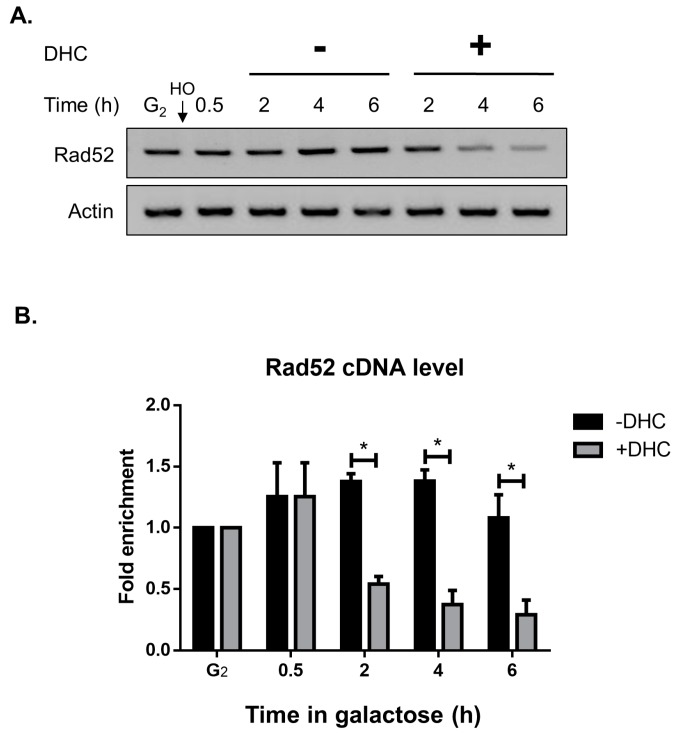
The cDNA levels of Rad52 are inhibited following DHC treatment. YMV045 cells were arrested at G2 with nocodazole, and HO endonuclease was induced by the addition of galactose to generate a DSB. After 30 min, the cultures were divided equally and treated with or without 5 mM DHC. Samples were processed for reverse transcription to generate cDNA and were analyzed by: (**A**) PCR; or (**B**) quantitative PCR. The error bars represent the standard deviations from three independent experiments. Asterisks (*) indicate significant differences between DHC-treated and untreated cells (* *p* < 0.05).

We noticed that the Rad52 protein levels were partially rescued by MG132 following DHC treatment, which suggests that DHC might also regulate Rad52 in addition to promoting its post-translational regulation. We then determined whether DHC inhibits Rad52 at the transcriptional level by analyzing changes in the Rad52 mRNA levels after DHC treatment. RT-PCR and RT-QPCR analyses revealed that the Rad52 mRNA levels were inhibited following DHC treatment (Figure 5). These data suggest that DHC decreases Rad52 protein expression by affecting both transcriptional and post-translational regulation. 

### 2.5. DHC Inhibits Rad52 Protein Levels through Its HDAC Inhibitor Activity

**Figure 6:**
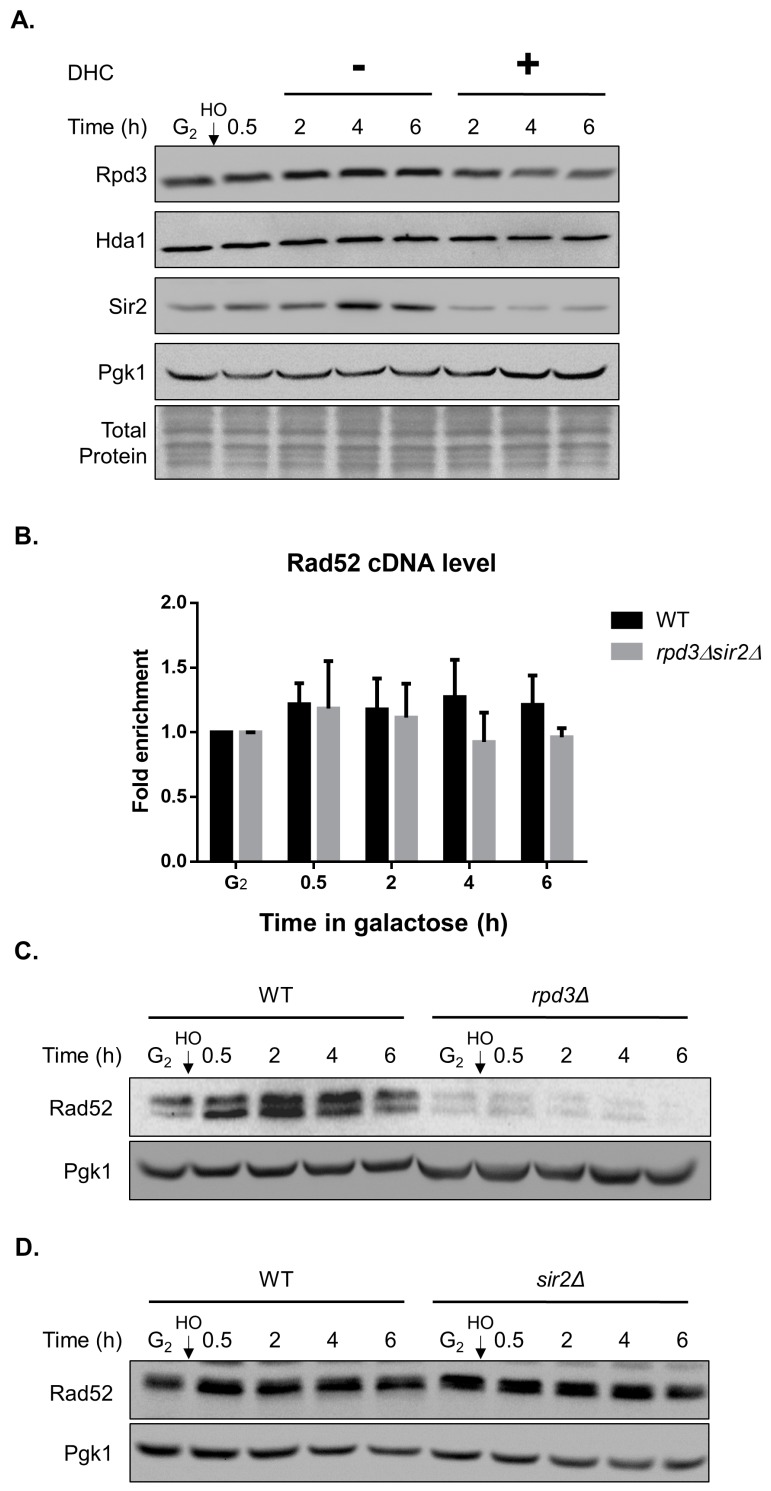
The inhibition of Rad52 protein expression by DHC is mimicked by *rpd3Δ* mutants. (**A**) The YMV045 cells were arrested at G2 with nocodazole, and HO endonuclease was induced by the addition of galactose at time 0 to generate a DSB. The culture was then split into two groups: −DHC and +DHC (5 mM DHC). Samples were processed for immunoblotting analysis using Rpd3, Sir2 and Hda1 antibodies; (**B**) A DSB was induced in *rpd3Δsir2Δ* double deletion cells (DHY002). Samples were processed for reverse transcription to generate cDNA and were quantitated by real-time QPCR. The error bars represent the standard deviations from three independent experiments; (**C**) *RAD52-HA* cells (YAY013) and *rpd3Δ* (YAY016) cells were arrested at G2 with nocodazole, and HO endonuclease was induced by the addition of galactose to generate a DSB. Samples were processed for immunoblotting analysis; (**D**) *RAD52-MYC* cells (YAY028) and *sir2Δ* (NKY001) cells were arrested at G2 with nocodazole, and HO endonuclease was induced by the addition of galactose to generate a DSB. Samples were processed for immunoblotting analysis.

A previous study showed that DHC is classified as a Sir2-HDAC (class III HDAC) inhibitor in yeast [28]. We thus first tested whether DHC inhibits Sir2 and other types of HDAC in yeast. The protein levels of Sir2 were impaired following DHC treatment, which is consistent with previous reports. In addition, our results provide the first demonstration that Rpd3, a class I HDAC, is also inhibited by DHC (Figure 6A). Reportedly, DDR is inhibited by HDAC inhibitors in an acetylation-dependent manner [29]. We then determined whether Rad52 is regulated by DHC through its HDAC inhibitor activity, and an RT-PCR analysis showed that the cDNA levels of Rad52 did not decrease in the *rpd3* or *sir2* single mutants (data not shown) and the *rpd3* *sir2* double mutant (Figure 6B). This result suggests that the transcriptional inhibition of Rad52 by DHC is independent of its HDAC activity. Moreover, by immunoblotting, we found that Rad52 protein expression was markedly decreased in the *rpd3* mutant, which mimics DHC treatment (Figure 6C), whereas Rad52 expression was not suppressed in the *sir2* mutant (Figure 6D). These data suggest that the translational inhibition of Rad52 by DHC is dependent on its HDAC activity for class I but not class III.

### 2.6. DHC Promotes Damage-Induced Apoptosis

**Figure 7:**
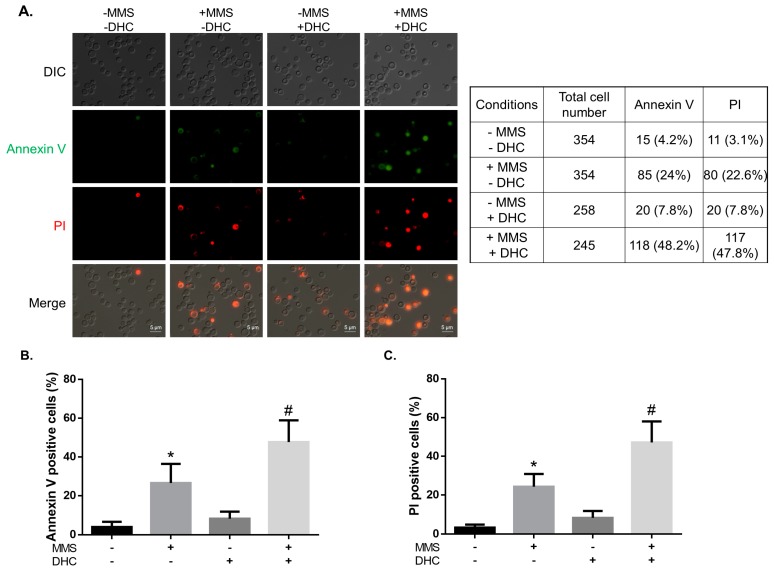
DHC synergistically stimulates apoptosis in response to DNA damage. (**A**) YMV045 cells were treated with 0.02% MMS for 1 h and then with or without 5 mM DHC for 3 h. The fluorescent signals of annexin V and PI were examined through fluorescence microscopy. The scale bars are 5 μm. The table shows the number of cells expressing annexin V or PI signals; (**B**,**C**) The panels show the percentage of fluorescence signals illustrated in (**A**). The error bars represent the standard deviations from three independent experiments. Asterisks (*) indicate significant differences between MMS-treated and untreated cells (* *p* < 0.05), and pound signs (#) indicate significant differences between MMS-treated and MMS + DHC-treated cells (# *p* < 0.05).

Our data showed that DHC increases the sensitivity of yeast cells to DNA damage. In addition, DHC-treated cells failed to repair DNA damage and postponed their cell cycle checkpoint. This phenomenon led us to investigate whether DHC-treated cells would subsequently induce apoptosis due to unrepaired DNA damage. We tested this hypothesis through annexin V and propidium iodide (PI) fluorescence staining for detecting early and late apoptosis. We monitored fluorescent staining in yeast cells treated with 0.02% MMS, 5 mM DHC and 0.02% MMS plus 5 mM DHC (Figure 7). The results showed that 24% and 22.6% of the MMS-treated yeast cells showed early apoptotic activity and late apoptotic activity, respectively. In contrast, 7.8% of the DHC-treated cells showed early apoptotic activity, and the same percentage of cells showed late apoptotic activity, which is consistent with previous reports that DHC slightly induces apoptosis [28]. In contrast, the percentage of early and late apoptotic cells increased markedly after treatment with both MMS and DHC (48.2% and 47.2%, respectively). These results demonstrate that DHC stimulates damage-induced apoptosis.

### 2.7. Damage-Induced Autophagy Is Impaired by DHC

**Figure 8:**
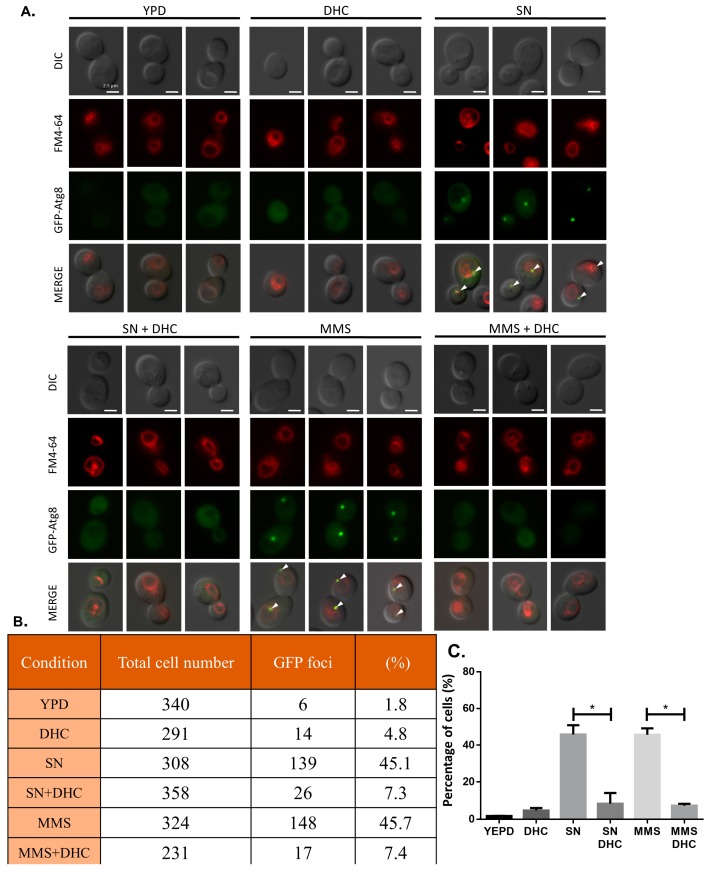
DHC inhibits nitrogen starvation and MMS-induced autophagy. (**A**) GFP-Atg8 (RLY004) cells were cultured in YPD, nitrogen starvation (SN) conditions, MMS (0.1%), DHC (5 mM), SN + DHC and MMS + DHC for 4 h. Samples were stained with FM4-64 and processed for fluorescence microscopy. DIC served as a control, and the scale bars are 2.5 μM. (**B**) The table shows the quantification of the fluorescence microscopy results shown in (**A**); (**C**) The panel shows the percentage of fluorescence signals. The error bars represent the standard deviations from at least three independent experiments. Asterisks (*) indicate significant differences between DHC-treated and untreated cells (* *p* < 0.05).

Autophagy has been shown to correlate with DDR and DNA repair [37,38]. We asked whether DHC influences autophagy after DDR. Atg8, a ubiquitin-like protein, facilitates cargo transport during autophagy in budding yeast. Therefore, the localization of Atg8 at a pre-autophagosomal structure (PAS) was analyzed as an indicator of autophagy [39,40]. We used a GFP-tagged Atg8 strain to detect yeast autophagy through fluorescence microscopy, and nitrogen starvation conditions were used as a positive control of autophagy. The results revealed that GFP-Atg8 localized to PAS following MMS treatment or under nitrogen starvation conditions, which is consistent with previous reports. Interestingly, the localization of Atg8 was abolished by DHC treatment (Figure 8). These data suggest that autophagy induced by MMS or nitrogen starvation conditions can be impaired by DHC. 

**Figure 9:**
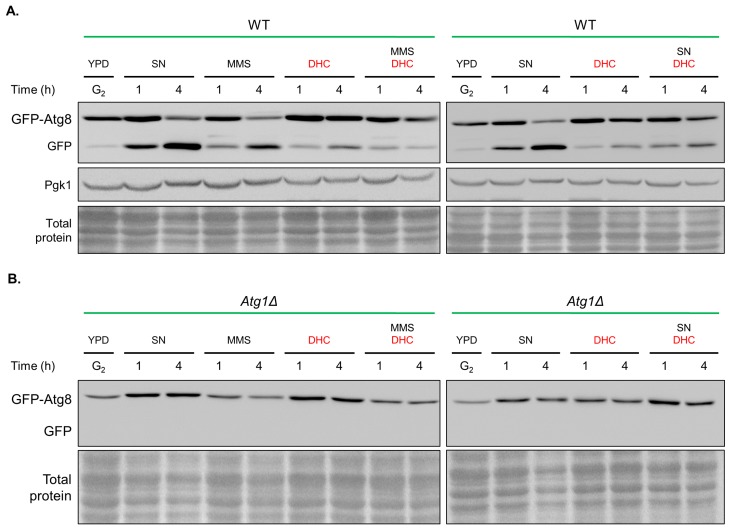
DHC inhibits autophagy in response to DNA damage. (**A**) GFP-Atg8 (RLY004) cells and GFP-Atg8 *atg1∆* (RLY005) cells were cultured in YPD, nitrogen starvation (SN) conditions, MMS (0.1%), DHC (5 mM), SN + DHC and MMS + DHC for 4 h. Samples were processed for Western blot using GFP antibodies. The Pgk1 protein level and total protein, as assessed through amido black staining, served as loading controls; (**B**) GFP-Atg8 *atg1∆* (RLY005) cells served as a negative control.

Upon delivery of a GFP-Atg8 molecule to the vacuole, the Atg8 moiety is rapidly degraded [39,41]. The appearance of GFP can thus be used to monitor autophagy through immunoblotting. Similarly, we found that the GFP levels were increased in the MMS-treated group and under nitrogen starvation conditions. However, the separation of GFP from GFP-Atg8 was decreased by combined treatment with DHC (Figure 9A). *atg1* mutants were used as negative controls with defective autophagy (Figure 9B). Taken together, the data demonstrate that MMS-induced autophagy and starvation-induced autophagy are impaired following DHC treatment.

## 3. Discussion

Targeting DNA repair pathways is an increasingly popular strategy for improving the efficacy of DNA damage-based cancer therapy. Although yeasts are not a good model for cancer research, we exploited the functional conservation of the DNA repair pathways between yeast and humans to mechanistically identify inhibitors of DNA repair proteins in yeast that could be extended to human use in the future. Our results show that DHC increases DNA damage sensitivity by suppressing DNA repair pathways, which suggests that DHC might have potential uses as a chemo- or radiosensitizer. Targeting human RAD52 represents a selective therapeutic approach for BRCA2-deficient cancers due to the synthetic lethality of RAD52 and BRCA2 [42]. In this study, we provide the first demonstration that the inhibitory activity of DHC toward the recombination protein Rad52 is critical for DHC-mediated sensitivity to DNA damage. Therefore, DHC can potentially be used for sensitizing BRCA2-deficient cancer cells. Our research supports the preclinical relevance of identifying molecular targets for DNA damage repair proteins, which will be of paramount importance in devising future therapeutic interventions. 

We noticed that wild-type cells are extremely sensitive to DHC combined with MMS but only slightly sensitive to DHC in combination with 4NQO or UV. It is known that MMS generates single- and double-strand breaks that can be repaired by HR, whereas 4NQO or UV generates cross-linked DNA that can be repaired by nucleotide excision repair (NER). Therefore, this phenomenon indicates that DHC might inhibit DNA repair pathways mainly by regulating HR-related protein(s). HDAC inhibitors, such as valproic acid (VPA) and curcumin, have been reported, and these can effectively inhibit HR repair and sensitize cells to DNA damage [29,30]. In this study, we also revealed that DHC, an effective HDAC inhibitor, inhibits DNA repair and increases DNA damage sensitivity in yeast. Reportedly, DHC inhibits NAD-dependent HDAC Sir2 in yeast [28]. In response to a DSB, SIRT1 (Sir2 in yeast) deacetylates NBS1, allowing its phosphorylation by ATM and recruitment of the MRE11–RAD50–NBS1 (MRN) complex, which is involved in the early stages of the DDR and is critical for mediating HR repair and DNA damage checkpoint activation [43,44,45]. However, the expression of Rad52 recombinase is not suppressed in *sir2* mutants; therefore, it is unlikely that DHC downregulates homologous recombination by Sir2 inhibition (Figure 6D). Whether DHC influences the DNA damage checkpoint through a Sir2-dependent pathway needs to be further tested. Surprisingly, our results showed that DHC attenuates DNA repair through Rad52 recombinase inhibition, which is a Rpd3-dependent process (Figure 6C). Robert et al. reported that VPA, an HDAC inhibitor with a different mechanism than DHC, inhibits both Rpd3 and Hda1 and triggers Sae2 degradation to suppress DNA end resection by increasing the acetylation of Sae2 [29]. In this study, we found that *rpd3* deletion alone mimics the effect of DHC treatment in Rad52 regulation, which suggests that DHC might inhibit Rad52 through its Rpd3 inhibitor activity. HDAC inhibition often results in the activation of gene transcription by hyperacetylating chromatin and loosening DNA structure [46,47], but the mechanism through which *rpd3* mutants and DHC treatment downregulate Rad52 needs further investigation. We also noticed that high concentrations of VPA (10 mM) are required to inhibit DDR in the paper by Robert et al. [29]. Similarly, 5 mM DHC is required for suppressing DDR due to the low permeability of drugs in yeast, but this requirement might not be a problem in mammalian cells [48]. 

In mammalian cells, low DNA damage levels transiently activate p53, and high DNA damage levels contribute to continuous p53 activation. Different levels of p53 activation might lead to differential expression of pro-survival and pro-apoptosis genes because the binding affinity of p53 to promoters is high for genes that are associated with cell cycle arrest and low for genes that are associated with apoptosis [49]. Although yeast does not have a protein homologous to human p53, a similar mechanism is still present [50,51,52,53]. We propose that cell death might be exacerbated if cells keep checkpoint signaling turned on when they face unrepairable DNA damage. Supporting this hypothesis, our findings reveal that DHC acts in a concerted manner to induce apoptosis when it is combined with DNA-damaging drugs (Figure 7). In addition to apoptosis, autophagy is also critical for cell survival and for protecting organisms from stresses [37,38,54]. The biological processes of autophagy and DDR have been linked, but the available knowledge regarding the mechanism through which DDR contributes to autophagy remains limited [55]. It was recently shown that the activation of Mec1-Ddc2 (ATR-ATRIP in human), Tel1 (ATM in human) and Rad53 (CHK2 in human) is required for damage-induced autophagy in yeast [56], suggesting that DHC might suppress damage-induced autophagy by inhibiting these checkpoint kinases. According to our findings (Figure 3), DHC did not inhibit MMS-induced autophagy by downregulating Mec1-Ddc2 or Rad53. However, it is still possible that DHC inhibits damage-induced autophagy through the downregulation of Tel1, but further work is needed to test this hypothesis. 

In conclusion, our data show that DHC sensitizes yeast to DNA damage by inhibiting DDR. In response to a DSB, DNA end resection proceeds normally, but homologous recombination is inhibited by the suppression of Rad52 by DHC. Consequently, cell death is synergistically promoted through increased damage-induced apoptosis.

## 4. Materials and Methods

### 4.1. Strains, Plasmids and Chemicals

All yeast strains used in this study are described in Table 1. DHC, 4NQO, protease inhibitor cocktail, nocodazole, MMS, G418 (G418 disulfate salt), TRIzol, BCP (1-Bromo-3-chloropropane), and annexin V-FITC apoptosis kit were purchased from Sigma-Aldrich. FM4-64 was purchased from Biotium. PI and HU were purchased from Santa Cruz Biotechnology. Dynabeads were purchased from Life Technologies (Carlsbad, CA, USA). The plasmid expressing the GFP fusion of Atg8 was a gift from Dr. Wei-Pang Huang (National Taiwan University, TPE, Taiwan).

### 4.2. HO Induction

Yeast cultures were grown for 12 to 14 h in yeast extract-peptone (YEP) medium containing lactic acid. DSBs were then induced by the addition of galactose (final concentration of 2%). Samples at time 0 were collected prior to the addition of galactose. Samples were collected for cutting and repair analysis, immunoblotting, RT-QPCR, or ChIP at the time points indicated in the figures.

### 4.3. Cutting and Repair Analysis

Cutting, repair and mating type switching of the HO lesion were measured by PCR amplification of genomic DNA templates collected from the above-described time courses using primers flanking the HO site as described previously [30]. PCR amplification prior to a DSB yields a 1.7-kb product, and analysis following repair by SSA yields a 3.0-kb product. Primers corresponding to the RAD3 gene were included in the multiplex PCR as an internal control. All primers used in this study are listed in Table 2.

### 4.4. DNA Damage Sensitivity Plate Assay

Yeast cells were diluted in five-fold increments and plated onto YPD, YEP medium containing 2% galactose or the indicated concentration of a DNA-damaging drug. The cells were grown at 30 °C for 3 days, and colony formation was recorded by photographs.

### 4.5. Immunoblotting

The preparation of yeast protein extract from TCA-treated cells was described previously [57]. The samples were separated by electrophoresis on a 10% SDS-polyacrylamide gel. The following primary antibodies were used for immunoblotting at a 1:1000 dilution: anti-Pgk1 (ab113678), anti-Rad53 (ab104232) and anti Y-H2A (ab15083) were obtained from Abcam; anti-Rpd3 (yC-19), anti-Hda1 (yC-20) and anti-Sir2 (yN-19) were obtained from Santa Cruz Biotechnology (Santa Cruz, CA, USA); and anti-HA (H9658) and anti-Myc (M4439) were obtained from Sigma-Aldrich. Anti-mouse (A9044), anti-rabbit (A0545) and anti-goat (A5420) HRP-linked secondary antibodies were obtained from Sigma-Aldrich (St. Louis, MO, USA) and used at a 1:100,000 dilution. Images were acquired with a Wealtec KETA-CL imaging system.

### 4.6. Chromatin Immunoprecipitation (ChIP) Assay

ChIP assays was performed as previously described [58]. Briefly, samples were treated with formaldehyde and subjected to chromatin shearing and immunoprecipitation to form protein-DNA complexes. Quantification of the DNA molecules present in immunoprecipitates was performed by qPCR using a Roche 480 instrument with the primers listed in Table 2. Anti-HA (Sigma-Aldrich, H9658), anti-Myc (Sigma-Aldrich, M4439) and anti-Rfa1 (Agrisera, Vannas, Sweden, ab15082) antibodies were used for ChIP.

### 4.7. RT-QPCR

RNA was extracted by the addition of TRIzol. BCP (Sigma-Aldrich, B9673) was added, and the samples were shaken for 30 s. The samples were maintained at room temperature for 3 min and then centrifuged (17,000× *g*, 15 min, 4 °C). The upper phase of the aqueous solution, which contained RNA, was collected in a fresh tube and precipitated by the addition of isopropanol. Samples were mixed by vortexing, maintained at RT for 10 min, and then centrifuged (17,000× *g*, 10 min, 4 °C). The supernatant was discarded, and the RNA pellet was washed in 75% ethanol by centrifugation (17,000× *g*, 5 min, 4 °C). The supernatant was discarded, and the pellet was resuspended in DEPC water. The total RNA concentrations were determined using a Nanodrop spectrophotometer (Thermo Scientific, Grand Island, NY, USA, 2000C). The RNA quality was analyzed by running a gel (1% agarose). DNA was digested with DNase (Promega, Madison, WI, USA, M6101), and total RNA was reverse transcribed using the Yeastern Reverse Transcription Kit (Yeastern, TPE, Taiwan, FYT501). 

Gene expression was analyzed by qPCR using a Roche 480 instrument with the primers listed in Table 2.

### 4.8. Fluorescence Microscopy

The yeast samples collected for apoptosis and autophagy assay were further analyzed by a Nikon ECLIPSE Ni-U plus fluorescence microscope equipped with 100× oil objectives. Images were acquired with a DS-U3 CCD camera and controlled using NIS-Element BR 4.0 software.

## Figures and Tables

**Table 1:** Genotypes of yeast strains used in this study.

Strain	Genotype	Source
YMV002	*MATα ho hml∆::ADE1 mata∆::ADE1 his4::-URA3-leu2-(Xho1 to Asp718)-his4 leu2::HOcs ade3::GAL::HO ade1 lys5 ura3-52*	James Haber
YMV037	*MATα ho hml∆::ADE1 mata∆::ADE1 his4::-URA3-leu2-(Xho1 to Asp718)-his4 leu2::HOcs ade3::GAL::HO ade1 lys5 ura3-52 rad52∆::HPH*	James Haber
YMV045	*MATα ho hml∆::ADE1 mata∆::hisG hmr∆::ade1 leu2::leu2 (Asp-718-SalI)-URA3-pBR322-HOcs ade3::GAL::HO ade1 lys5 ura3-52 trp1*	James Haber
YMV046	*MATα ho hml∆::ADE1 mata∆::hisG hmr∆::ADE1 leu2:HOcs ade3::GAL::HO ade1 lys5 ura3-52 trp1 rad52∆::HPH* (hygro)	James Haber
YMV 057	*MATα ho hml∆::ADE1 mata∆::hisG hmr∆::ade1 leu2::leu2 (Asp-718-SalI)-URA3-pBR322-HOcs ade3::GAL::HO ade1 lys5 ura3-52 trp1 srs2::HPH*	James Haber
BY4741	*MATa his3∆ leu2∆ met15∆ ura3∆*	This study
BY4741-atg1	*MATa his3∆ leu2∆ met15∆ ura3∆ atg1∆::KAN*	This study
BY4741-atg8	*MATa his3∆ leu2∆ met15∆ ura3∆ atg8∆::KAN*	This study
BY4741-gcn5	*MATa his3∆ leu2∆ met15∆ ura3∆ gcn5∆::KAN*	This study
BY4741-hat1	*MATa his3∆ leu2∆ met15∆ ura3∆ hat1∆::KAN*	This study
BY4741-hda1	*MATa his3∆ leu2∆ met15∆ ura3∆ hda1∆::KAN*	This study
BY4741-rad6	*MATa his3∆ leu2∆ met15∆ ura3∆ rad6∆::KAN*	This study
BY4741-rpd3	*MATa his3∆ leu2∆ met15∆ ura3∆ rpd3∆::KAN*	This study
BY4741-sir2	*MATa his3∆ leu2∆ met15∆ ura3∆ sir2∆::KAN*	This study
BY4741-rtt109	*MATa his3∆ leu2∆ met15∆ ura3∆ rtt109∆::KAN*	This study
RLY001	*MATα ho hml∆::ADE1 mata∆::hisG hmr∆::ade1 leu2::leu2 (Asp-718-SalI)-URA3-pBR322-HOcs ade3::GAL::HO ade1 lys5 ura3-52 trp1 (trp1::hisG) DDC2-MYC::KAN*	This study
YAY012	*MATα ho hml∆::ADE1 mata∆::hisG hmr∆::ade1 leu2::leu2(Asp-718-SalI)-URA3-pBR322-HOcs ade3::GAL::HO ade1 lys5 ura3-52 trp1 (trp1::hisG) rpd3∆::KAN*	This study
YAY013	*MATα ho hml∆::ADE1 mata∆::hisG hmr∆::ade1 leu2::leu2 (Asp-718-SalI)-URA3-pBR322-HOcs ade3::GAL::HO ade1 lys5 ura3-52 trp1 (trp1::hisG) HA-RAD52::KAN*	This study
YAY016	*ho hml∆::ADE1 mata∆::hisG hmr∆::ade1 leu2::leu2(Asp-718-SalI)-URA3-pBR322-HOcs ade3::GAL::HO ade1 lys5 ura3-52 trp1 (trp1::hisG?) HA-Rad52::KAN Rpd3::TRP*	This study
YAY028	*MATα ho hml∆::ADE1 mata∆::hisG hmr∆::ade1 leu2::leu2(Asp-718-SalI)-URA3-pBR322-HOcs ade3::GAL::HO ade1 lys5 ura3-52 trp1 (trp1::hisG) MYC-RAD52::TRP*	This study
NKY001	*ho hml∆::ADE1 mata∆::hisG hmr∆::ade1 leu2::leu2(Asp-718-SalI)-URA3-pBR322-HOcs ade3::GAL::HO ade1 lys5 ura3-52 trp1 (trp1::hisG?) Myc-Rad52::TRP Sir2∆::KAN*	This study
RLY004	BY4741-atg8 transformed with PRS416 GFP-Atg8 in URA drop media	This study
RLY005	BY4741-atg1 transformed with PRS416 GFP-Atg8 in URA drop media	This study
RLY006	*MATαho hml∆::ADE1 mata∆::hisG hmr∆::ade1 leu2::leu2(Asp-718-SalI)-URA3-pBR322-HOcs ade3::GAL::HO ade1 lys5 ura3-52 trp1 (trp1::hisG) MYC-RAD52::TRP sem1::KAN*	This study
DHY001	*MATα ho hml∆::ADE1 mata∆::hisG hmr∆::ade1 leu2::leu2(Asp-718-SalI)-URA3-pBR322-HOcs ade3::GAL::HO ade1 lys5 ura3-52 trp1 (trp1::hisG?) Sir2::KAN*	This study

**Table 2:** Primers used in this study.

Primer	Sequence
SSA1	CCGCTGAACATACCACGTTG
SSA2	CACTTCCAGATGAGGCGCTG
SSA3	TGAACTCTGGTGTCTTTTAG
RAD3A	GATAAGATTGCGACAAAAGAGGATA
RAD3D	GTGGGACGAGACGTTTAGATAGTAA
HO-F	CCAAATCTGATGGAAGAATGGG
HO-R	CCGCTGAACATACCACGTTG
SMC2-F	ATCACTGATTGAAGAGGCAGC
SMC2-R	TACGAGTCTCACCGTTCTCCA
Rad52-int-RNA-F	TGGCTGGTCTACGGAGGTAA
Rad52-int-RNA-R	GCGGTGGTCATCGTTTTGTC
Rad52-int-QPCR-F	TCAAGTACCGCGTGAAACCA
Rad52-int-QPCR-R	CGATCTTTGTTGCGGAACGG
Actin-int-F	TACGTTTCCATCCAAGCCGT
Actin-int-R	CGGCAGATTCCAAACCCAAA
